# FNDC5/Irisin System in Neuroinflammation and Neurodegenerative Diseases: Update and Novel Perspective

**DOI:** 10.3390/ijms22041605

**Published:** 2021-02-05

**Authors:** Patrizia Pignataro, Manuela Dicarlo, Roberta Zerlotin, Chiara Zecca, Maria Teresa Dell’Abate, Cinzia Buccoliero, Giancarlo Logroscino, Silvia Colucci, Maria Grano

**Affiliations:** 1 Department of Basic Medical Sciences, Neuroscience and Sense Organs, University of Bari, 70124 Bari, Italy; patrizia.pignataro@uniba.it (P.P.); manuela.dicarlo@uniba.it (M.D.); giancarlo.logroscino@uniba.it (G.L.); silviaconcetta.colucci@uniba.it (S.C.); 2 Department of Emergency and Organ Transplantation, University of Bari, 70124 Bari, Italy; roberta_zerlotin@libero.it (R.Z.); cinzia.buccoliero@uniba.it (C.B.); 3 Center for Neurodegenerative Diseases and the Aging Brain, Department of Clinical Research in Neurology, University of Bari, “Pia Fondazione Card G. Panico” Hospital Tricase, 73039 Lecce, Italy; chiarazecca.cz@gmail.com (C.Z.); dellabatemariateresa@gmail.com (M.T.D.)

**Keywords:** irisin, FNDC5, neuroinflammation, neurodegeneration

## Abstract

Irisin, the circulating peptide originating from fibronectin type III domain-containing protein 5 (FNDC5), is mainly expressed by muscle fibers under peroxisome proliferator-activated receptor gamma coactivator 1-alpha (PGC1α) control during exercise. In addition to several beneficial effects on health, physical activity positively affects nervous system functioning, particularly the hippocampus, resulting in amelioration of cognition impairments. Recently, FNDC5/irisin detection in hippocampal neurons and the presence of irisin in the cerebrospinal fluid opened a new intriguing chapter in irisin history. Interestingly, in the hippocampus of mice, exercise increases FNDC5 levels and upregulates brain-derived neurotrophic factor (BDNF) expression. BDNF, displaying neuroprotection and anti-inflammatory effects, is mainly produced by microglia and astrocytes. In this review, we discuss how these glial cells can morphologically and functionally switch during neuroinflammation by modulating the expression of a plethora of neuroprotective or neurotoxic factors. We also focus on studies investigating the irisin role in neurodegenerative diseases (ND). The emerging involvement of irisin as a mediator of the multiple positive effects of exercise on the brain needs further studies to better deepen this issue and the potential use in therapeutic approaches for neuroinflammation and ND.

## 1. Overview of FNDC5/Irisin System

Irisin is one of the novel exercise-induced myokines firstly described by Boström and colleagues in 2012 [1].

**Figure 1:**
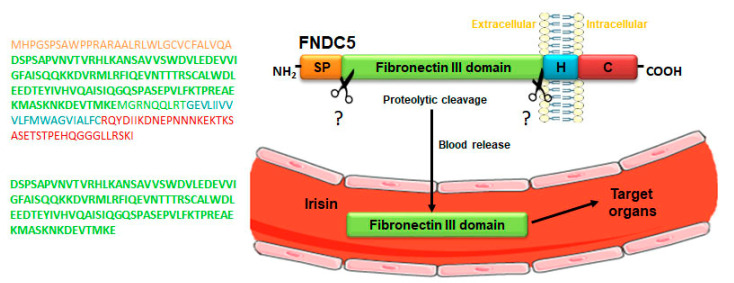
Mechanism of irisin release into the blood. Schematic structure of irisin precursor fibronectin type III domain-containing protein 5 (FNDC5) and its amino acid sequence (upper panel). FNDC5 cleavage, by a yet unknown enzyme at levels of the extracellular domain, gives rise to the release of irisin into the blood, a peptide consisting of a sequence of 112 amino acids (lower panel). Abbreviations: SP, signal peptide; H, hydrophobic domain; C, cytoplasmic domain. Symbols: ? indicates the unknown enzyme.

Irisin is produced from a transmembrane glycoprotein, named fibronectin type III domain-containing protein 5 (FNDC5), which is cleaved in its extracellular domain by a yet unidentified enzyme, resulting in the release of a 112 amino acids polypeptide into the bloodstream (Figure 1). Interestingly, the discovery of the circulating irisin was subsequent to that of its precursor. In 2002, two different research groups during their cloning studies in mice demonstrated the expression of irisin precursor FNDC5 as peroxisomal protein (PeP) and fibronectin type III repeat-containing protein 2 (FRCP2). In particular, Ferrer-Martínez et al. found PeP in the peroxisome matrix of various cell types, and Teufel et al. discovered the gene encoding FRCP2 while studying different fibronectin type III domains [2,3]. Both of these researchers identified irisin precursor expression in the murine brain. A robust PeP expression at the mRNA level was shown, by Northern blot analyses, only in adult mice brain, while it was absent during mouse development [2]. Conversely, the expression of *Frcp2* was detected in both embryonic and adult brain tissue, suggesting its specific role during head development and for adult brain functioning [3]. Subsequently, the initial existence of the two different molecules, PeP and FRCP2, gave rise to the recognition of the same product renamed FNDC5. The expression of this membrane glycoprotein is regulated by the transcriptional coactivator peroxisome proliferator-activated receptor gamma co-activator-1α (PGC-1α), which, in turn, is induced by physical activity [1].

Comparative studies demonstrated that *Fndc5* is a well-preserved gene among species, and 100% of identity has been found between human and murine irisin sequences [1]. Conversely, only the human FNDC5 gene displays an uncommon ATA start codon [4], and this was considered as a “null mutation” that would prevent human irisin production and its release into the blood [5,6]. In humans, bioinformatics analyses have identified the existence of three isoforms for FNDC5 mRNA that were all expressed during the neural differentiation process [7]. Furthermore, high expression levels of FNDC5 were detected in human fetal brain and spinal cord tissues, suggesting the involvement of this gene in neural tube development [7]. In rodents, FNDC5 expression was revealed in various cerebral regions, including cerebellar Purkinje cells [8], hypothalamus [9], and hippocampus [10].

Jedrychowski et al. detected a significant increase of irisin concentration in the plasma of exercised subjects with respect to the sedentary ones (4.3 ng/mL versus 3.6 ng/mL) using a targeted mass spectrometry approach [4]. Besides physical activity, several factors, such as diet, obesity, metabolic diseases, as well as treatments for such conditions, and various other pathological disorders (chronic renal failure, hypothyroidism, musculoskeletal, and neurodegenerative diseases) affect the circulating irisin levels [11,12,13,14]. Very recently, a reduced irisin serum level was found in patients with age-related bone diseases with respect to healthy subjects [15]. By means of ELISA assays and quantitative mass spectrometry analyses, irisin was also found in human cerebrospinal fluid (CSF) [16,17,18]. Recently, Ruan et al. detected the irisin circulating levels in CSF at a concentration of about 0.26–1.86 ng/mL in old male subjects (over 80 years of age) with various diseases [17]. Moreover, an age-related increase in irisin levels in the CSF of healthy humans was found [18].

In the investigation of irisin tissue targets, Boström et al. were the first to recognize the irisin effect on adipose tissue, showing its ability in inducing white adipose tissue browning (i.e., the transdifferentiation of white adipocytes into brown-like fat cells) via enhancing mitochondrial uncoupling protein 1 (UCP1) expression [1]. Succeeding evidence highlighted more wide-ranging effects of irisin on other tissues as its role in regulating energy metabolism. In particular, the myokine promotes glucose and lipid uptake by skeletal muscles [11,19], increases hepatic glucose and lipid metabolism [20,21], displaying beneficial effects on metabolic disorders, such as diabetes [11,22].

Irisin displayed a noteworthy anabolic role on bone by the stimulation of osteoblast differentiation and activity [23]. This finding paved the way towards the investigation of irisin effects on hindlimb suspended mice, a widely recognized disuse-induced osteo-sarcopenic model characterized by the reduction of bone and muscle mass. Noticeably, in these mice, irisin positive action was shown on the musculoskeletal system, resulting in the simultaneous restoration of both muscle and bone mass loss [24]. A similar effect on osteoblasts was reported in a 3D in vitro model placed in a microgravity environment [25]. In addition, the last studies evidenced also a new intriguing role of irisin in delaying osteoblast senescence by the downregulation of *p21*, a well-known negative cell cycle regulator [15]. Besides osteoblasts, the osteocytes, the most abundant bone cell population, are irisin targets as the myokine improved their functions and displayed anti-apoptotic effects [26].

At present, the complete identification of irisin receptors is still lacking, but Kim et al. proposed integrins, specifically αV/β5, as its receptors on murine adipocytes and osteocytes [27]. However, the binding affinity of irisin to other integrin complexes, as well as the ability of integrin αV/β5 to interact with other ligands, hindered the comprehension of irisin specific effects in vivo.

The identification of the irisin receptor is still a challenge; however, the intracellular signaling pathways activated by irisin have been partially elucidated. Irisin principally exerts its biological functions via mitogen-activated protein kinases (MAPK) signaling; nonetheless, 5′ adenosine monophosphate-activated protein kinase (AMPK), phosphatidylinositol 3-kinase/protein kinase B (PI3K/AKT), and signal transducer and activator of transcription 3 (STAT3)/Snail pathways are also involved in additional effects [28]. In the brain, recent studies demonstrated that the irisin neuroprotective role seems to be mediated by both STAT3 and cyclic adenosine 3′,5′-monophosphate/protein kinase A/cAMP response element-binding protein (cAMP/PKA/CREB) signaling pathways [29,30].

Overall, in less than a decade since the discovery of irisin, a large number of studies have revealed that irisin induces a plethora of benefits on human health. In vivo treatment with rec-irisin or the peripheral delivery of FNDC5 with adenoviral vectors evidenced that its beneficial effects recapitulate those of regular exercise on many organs, including brain health, in which the armament of the anti-inflammatory strategy involves the exercise-mimetic hormone irisin [1,10,23,30].

## 2. Physical Activity and FNDC5/Irisin in Neuroinflammation

The paradigm that physical activity displays beneficial effects on human health as a non-pharmacological approach is widely recognized. Its benefits are exerted not only on skeletal muscle but even on multiple non-skeletal targets. Indeed, exercise contributes to reducing adiposity and the risk of insulin resistance, displays beneficial effects on cardiovascular diseases, hypertension, bone and joint diseases, and metabolic disorders. Interestingly, exercise increases cerebral blood flow and exerts a plethora of positive effects on the brain, improving cognitive functions, such as memory and attention, and overall neuronal plasticity [31]. These latter effects were mostly detected in elderly subjects and in patients suffering from psychiatric and neurodegenerative diseases. Hippocampus, particularly the dentate gyrus, is the brain region mainly targeted by the positive impact of exercise [10,32,33]. Studies in humans demonstrated an increase in volume and improvement of functional connectivity in the hippocampus of elderly subjects, resulting in memory progress [34]. Studies in mice showed that the beneficial effects of exercise on hippocampal and synaptic function are due to the activation of metastasis-suppressor 1-like (Mtss1L) transcription factor in the dentate gyrus [35].

Although the mechanism responsible for these beneficial effects are at present not completely elucidated, the molecular mediators in charge seem to be two groups of factors: neurotrophins, produced locally in the brain, and myokines, released by muscle, which can equally or synergistically exert positive responses [36,37].

Neurotrophins, a family of proteins crucial for neuron development, growth, and plasticity, represent the supervisors of brain cell health, and their levels are altered in pathological conditions, i.e., neuroinflammation. Neuroinflammation, frequently associated with neurodegenerative diseases, is linked to several factors, such as infections, toxic metabolites, autoimmunity, aging, traumatic brain, or spinal cord injury, etc. [38]. In the presence of these pathological insults, microglia, the main immune cell type of central nervous system (CNS), are the first responders of neuro-inflammatory processes through the activation of phagocytosis and secretion of a variety of soluble factors [39,40]. These resident immune cells, arising from myeloid progenitors in the embryonic yolk sac, express several pattern-recognition receptors (PRRs) that can recognize pathogen-associated molecular patterns (PAMPs) or tissue damage-associated molecular patterns (DAMPs) [39]. Of note, microglia are highly dynamic cells as they can acquire different morphological and functional features. In their steady-state, microglia display a ramified shape with multiple and thin processes by which they constantly monitor the CNS parenchyma [39]. In response to inflammatory stimuli or extended injuries, microglia switch to an amoeboid morphology with enhanced phagocytic activity and pro-inflammatory cytokines production [interleukin (IL)-1 beta (IL-1β), IL-6, and tumor necrosis factor (TNF)α] [39,41]. In addition, microglia also release specific chemokines [42], which are chemoattractants for peripheral immune cells migrating into CNS to cooperate in the resolution of neuroinflammatory response, reactive oxygen species (ROS), and secondary messengers (nitric oxide, NO, and prostaglandins) [38].

If microglia activity is impaired, acute or chronic inflammatory processes can persist in the CNS, leading to irreversible outcomes, i.e., neurodegeneration [39].

Besides microglia, other glial cells, such as astrocytes, can contribute to the local immune responses induced by different insults. Astrocytes are the most abundant glial cell population in the adult CNS, controlling the blood–brain barrier integrity, the neuronal function through the production of neurotrophic factors, the extracellular balance of ions, etc. [42]. Similar to microglia, astrocytes are immunocompetent cells as they can take part in the regulation of innate and adaptive immune responses in the injured CNS [43]. Astrocytes express many receptors involved in innate immunity [44], and when activated by inflammatory stimuli, these cells secrete soluble mediators (IL-1β, TNFα, NO, etc.), triggering innate and/or adaptive immune responses [42].

Microglia and astrocytes can strictly cooperate in the inflammatory process. Indeed, the inflammatory factors released by microglia can induce pro-inflammatory astrocyte activation [42,45].

Interestingly, both microglia and astrocytes can display pro-inflammatory (or neurotoxic) and neuroprotective phenotype [42]. According to an early classification, microglia may be divided into M1 (classical activation) and M2 (alternative activation) phenotypes using the same paradigm of macrophage activation [39]. The first corresponds to the above-mentioned pro-inflammatory state that is induced by the concurrent activation of toll-like receptors (TLRs) and interferon-γ (IFN-γ) signaling pathways, while the second represents the anti-inflammatory condition linked to neuroprotection and tissue healing [39]. Astrocytes also may be classified in A1 (pro-inflammatory) and A2 (anti-inflammatory) reactive astrocytes on the basis of their polarization status. However, M1/M2 and/or A1/A2 paradigms are not always acceptable as omic studies have shown multiple activated phenotypes in vivo [39,46].

The response of microglia and astrocytes to peripheral factors (pro- and anti-inflammatory) proves that there is a clear interaction between the immune system and CNS, and, furthermore, as physical activity has been recently considered an anti-inflammatory agent, exercise, brain, and immune responses are also linked. Indeed, in response to muscle activity, the release of some myokines can affect microglial activation, such as the anti-inflammatory cytokine IL-10, which exerts an inhibitory effect on the microglial activation [47].

IL-6, the principal myokine that appears in the circulation during exercise [48], can display an indirect anti-inflammatory effect through the upregulation of IL-10 expression [49]. Consistent with this finding, in Alzheimer’s Disease (AD) and Parkinson’s Disease (PD) patients, a decrease of IL-6 levels has been detected [50,51]. However, the biological relevance of exercise-derived IL-6 is still hotly debated since it has been demonstrated that its increase is transient and downregulated by constant training [48]. In addition, IL-6 increase is frequently associated with chronic low-grade inflammation [52], and, recently, a longitudinal study proposed IL-6 as a predictor marker of cognitive decline in late midlife [53]. Of note, while other pro-inflammatory cytokines (IL-1 and TNF-α) induce the further expression of inflammatory mediators (NO or matrix metalloproteinases), muscle-derived IL-6 shows a peculiar behavior through its beneficial role on regenerative processes as well as on metabolism regulation [51,54]. Thus, the comprehension of IL-6 effects is still far from being completely elucidated since this myokine can exert a pro-inflammatory or anti-inflammatory role depending on the activation of trans-signaling via its soluble receptor or classic-signaling by its membrane-bound receptor, respectively [51].

A relevant additional role of exercise on neuroinflammation is the ability to upregulate the brain-derived neurotrophic factor (BDNF) levels. Interestingly, BDNF could be the linkage molecule between brain health and exercise, particularly because its production is linked to FNDC5/irisin expression. It has been demonstrated that in the hippocampus of mice, elevated FNDC5 expression was induced by endurance exercise under the control of PGC-1α, and, in this condition, a neuroprotective gene program, including the *Bdnf* gene, was activated [10]. BDNF, mainly released by microglia and astrocytes, plays a pivotal role in neuron formation, survival, and plasticity, contributing to memory and learning. It has been observed that neurons are also a BDNF source [10]. Indeed, forced expression of FNDC5 via adenoviral transfection or exogenous administration of rec-irisin increased *Bdnf* gene expression in primary cortical and hippocampal neurons, regardless of glial cells. On the contrary, loss of function of FNDC5 resulted in BDNF reduction [10]. Importantly, it has been shown that BDNF regulated FNDC5 levels according to a homeostatic feedback loop as rec-BDNF administration reduced *Fndc5* gene expression, while this effect was attenuated by the BDNF receptor blockage [10].

The anti-inflammatory effect of BDNF through microglia activation may be exerted by means of different pathways. Firstly, it could bind to its receptor tropomyosin receptor kinase B (TrkB), inducing the activation of extracellular-signal-regulated kinase (ERK) and the phosphorylation of CREB, which, in turn, can impede nuclear factor-kappa light chain enhancer of activated B cells (NF-κB) activity, hindering anti-inflammatory gene transcription [55,56]. Moreover, BDNF might act through Akt signaling, which blocks glycogen synthase kinase 3 (GSK-3) activity, thus reducing the activation of NF-κB and inducing CREB activation [57]. Finally, BDNF is also able to control mitogen-activated protein kinase phosphatase 1 (MKP-1), inducing the reduction of p38 and c-Jun N-terminal kinase (JNK) phosphorylation [58,59,60].

Due to the evidence that the circulating irisin, although with a not yet well-clarified mechanism, can reach the brain and enhance BDNF expression [10], this myokine might be the molecule inducing neuroprotection responses. Consistent with this, in 2017, studies in mice demonstrated that irisin, through the activation of anti-apoptotic signals, as AKT and ERK1/2 pathways, was involved in the neuroprotective effect of physical exercise against cerebral ischemia [61]. In the same year, the irisin engagement in the inhibition of the inflammatory pathway, such as reactive-oxygen species-Nod-like receptor pyrin-3 (ROS-NLRP3) signal, suggesting irisin as a possible treatment in ischemic stroke was shown in neuron cells [62]. In 2018, in vivo findings highlighted that irisin intrathecal injection increased pain threshold [63], and in vitro data showed that irisin supplementation in media collected from astrocytes protected neurons from β-amyloid toxicity [64]. In astrocytes, irisin could also decrease the release of IL-6 and IL-1β cytokines, the expression of cyclooxygenase-2 (COX-2) pro-inflammatory mediator, and phosphorylation of AKT, suggesting an important role of irisin in neurodegenerative diseases [64]. In addition to all this, there are very recent studies that showed a protective role of irisin via the activation of autophagy. Autophagy, as a potent cell strategy for protecting against inflammation, has been proposed as a mechanism by which physical exercise mediates its beneficial effects on health. In accordance with this hypothesis, several reports showed that exercise could trigger autophagy in different tissues and organs [65]. Importantly, in the AD mice model, the genetically-induced hyperactivation of autophagy, by knock-in of a point mutation in the gene *Beclin/Becn1*, which is essential in autophagy, resulted in the decrease of amyloid accumulation and limitation of cognitive decline. Likewise, in these mice models, physiological autophagy was induced by physical activity, resulting in protective effects on amyloid accumulation and memory, thereby exerting a protective effect in the brain [66]. In support of autophagy’s importance as an anti-inflammatory strategy and the role of irisin on autophagy, there is the very recent work of Xin and Lu of March 2020 [67]. These authors showed that mitophagy, a peculiar autophagic process that selectively involves mitochondria, was induced by irisin through the upregulation of the expression of optic atrophy 1 (*Opa1*). In particular, irisin activated *Opa1*-induced mitophagy, and this event preserved mitochondrial function, decreased oxidative stress, and inhibited apoptosis of cardiomyocytes following myocardial infarction [67]. Therefore, this irisin shielding mechanism [65] can be added to the other described effects of the myokine, underlying the importance of irisin in alleviating neuroinflammatory responses and neurodegenerative diseases.

## 3. Irisin and Neurodegenerative Diseases

As reported in the previous sections, FNDC5/irisin is highly expressed in the brain [8] and, through the modulation of BDNF levels [10], could be involved in hippocampal neurogenesis, affecting memory and cognition.

Over the last years, some papers, although still few and in a limited number of animal models, carried out an important role of irisin in neurodegeneration and in the attenuation of neurodegenerative processes. However, the mechanism and the possible role of irisin in neuroinflammation, which often is linked with neurodegeneration [68], has not yet been investigated.

Neuroinflammation plays a key role in the onset of neurodegenerative diseases as well as in their pathogenesis. In turn, neurodegeneration triggers neuroinflammatory processes, creating a detrimental vicious circle [68].

As described in the previous paragraph, the neuroinflammatory response primarily protects the brain from pathogenic injuries through their removal and the induction of tissue repair [39]. However, a prolonged inflammatory process, due to an abnormal release of neurotoxic factors and the persistence of activated glial cells, can negatively affect neuronal survival, inducing neurodegenerative processes [69]. Genetic factors, abnormal protein aggregation, or environmental agents (infections, trauma, and drugs) seem to be crucial in the permanent activation of microglia and astrocytes [42].

It has been demonstrated that peripheral inflammation also contributes to neuroinflammation by increasing blood–brain barrier permeability, which determines the further infiltration of the peripheral immune and inflammatory cells in the nervous tissues [68].

Besides the dysregulation of the inflammatory responses, the abnormal protein accumulation in the cytoplasm of neurons and/or in the extracellular space can be responsible for neuroinflammation associated with neurodegenerative disorders. Indeed, many of these diseases share the accumulation of misfolded proteins [70].

It could be of great interest to investigate the role of irisin on cells involved in neuroinflammation, since according to the papers we have discussed below, irisin seems to have a role in attenuating the development and progression of some neurodegenerative diseases, such as Alzheimer’s disease (AD), Parkinson’s disease (PD), and amyotrophic lateral sclerosis (ALS).

Literature data indicate that irisin can exert an anti-inflammatory effect in other tissues by acting on the cascade of inflammatory cytokines. In particular, by targeting different pathways, irisin has been shown to protect various tissues through anti-inflammatory, anti-oxidative, and anti-apoptotic effects [71]. Furthermore, very recently, irisin has been implicated in the protection against spinal cord injury in rats, targeting the oxidative stress-related markers via AMPK-NF-kB [72].

*Irisin and AD*. AD is a progressive neurological disease that predominantly impairs memory due to alterations and loss of synapses in a selected area of the brain, primarily the hippocampus [73,74,75].

The synaptic dysfunction is caused by the accumulation of hyperphosphorylated Tau (pTau) protein, induced by toxic amyloid-beta (Aβ) peptide. These aggregates attract and activate glial cells, inducing an inflammatory response through the release of neuroinflammatory factors that contribute to neurodegeneration [68].

The Aβ peptide is generated by β-, α-, and γ-secretase cleavage of the amyloid precursor protein (APP), leading to the formation of Aβ fragments, including Aβ40 and Aβ42, which accumulate in the cytoplasm of neurons and in the extracellular space [76,77,78]. The cleavage of β-secretase represents the primary step in the Aβ polypeptide formation and causes the amyloid cascade that leads to neurodegeneration in AD [79]. In fact, the β-secretase enzyme, termed beta-site amyloid precursor protein cleaving enzyme 1 (BACE1), in the brain cortex of AD patients is significantly increased [80]. Therefore, BACE1 could be a drug target for lowering cerebral Aβ levels in the treatment and/or prevention of AD [81].

A recent in vitro study, by means of biochemical and in silico simulation assay, showed the possible involvement of FNDC5/irisin in the mechanisms of Aβ generation [82]. In particular, the authors showed that APP/FNDC5 interaction, occurring by the binding of FNDC5 to a specific domain between β- and α-secretase cleavage sites of APP, could reduce Aβ formation, leading to a decreased secretion of both Aβ40 and Aβ42 peptides in the culture media. 

These results could be of great clinical relevance if studies in human and mouse models will provide a role of FNDC5 in the decrease of Aβ accumulation. This could imply the involvement of FNDC5 in the prevention of AD, acting in the initial phases of the disease development when Aβ accumulation begins and before the onset of dementia. Indeed, when AD is in the clinical stage, Aβ and tau accumulations have already induced extensive neuronal damage in multiple areas of the brain and could be too late for the possible FNDC5 role as of any other compound in AD treatment [82]. In vivo studies performed in animal models and in humans by Lourenco et al. [10,30] demonstrated that irisin levels were reduced in the cerebrospinal fluid (CSF) of AD mice and patients. In particular, downregulation of brain FNDC5/irisin in mice compromises long-term potentiation and novel object recognition memory, while the improvement of FNDC5/irisin brain levels restores synaptic plasticity and memory. Furthermore, forced peripheral expression of FNDC5/irisin rescues memory impairment. These significant results indicate that irisin could play a role in memory and suggest that its reduction could contribute to the cognitive decline [30].

In agreement with these data, another study, recently published by the same authors, demonstrated that irisin CSF levels in AD patients positively correlated with BDNF, Aβ42, and cognitive performance [83]. Despite the importance of this study, the limitation is due to the low number of patients since the investigation was performed on a cohort of 39 patients, subdivided into 25 non-demented controls and 14 demented AD patients.

A further human study by Küster et al. showed a significant association of irisin with episodic memory and global cognition in subjects at risk of dementia [84].

Later, it was also demonstrated, in humans, that irisin levels could be one of the factors influencing neurocognitive deficits in obese patients at genetic risk for AD [85].

Basically, the importance of irisin in neurodegenerative diseases and the possible crosstalk between peripheral levels of irisin and its role in the CNS are increasingly emerging, but further studies are needed. It would be important to study the spectrum of clinical cognitive decline before the dementia stage, focusing on mild cognitive impairment [86] and subjective cognitive decline [87].

Another consideration is that AD pathology with the deposition of amyloid and tau begins several years before the clinical onset and that this phase of the disease is identifiable through the use of appropriate biomarkers [88] and is possible to identify three subsequent stages. It is also possible to identify biological AD, restricting cases to subjects with changes in AD biomarkers. The study of irisin within the early stages of the AD biological pathway would be extremely innovative.

*Irisin and PD*. PD is a motor disorder due to the alteration and death of dopaminergic neurons of the substantia nigra of the brain. It is characterized by an accumulation of α-synuclein cytosolic protein, the main component of Lewy bodies and involved in the pathogenesis of PD [89]. The mechanisms through which neurons become damaged are still unclear. However, one of the causes could be represented by neuroinflammation. In fact, dopaminergic neurons seem to be more sensitive to neurotoxic factors released by the activated glial cells [68].

In 2019, Zarbakhsh et al. created a rat model of PD by intranasal injection of 1-methyl-4-phenyl-1,2,3,6-tetrahydropyridine (MPTP) [90]. They demonstrated that the simultaneous treatment of these rats with irisin and bone marrow stromal cells (BMSCs) resulted in the protection of the dopaminergic neurons from apoptosis and degeneration. In particular, they found that in the brain of these animals, irisin induced the migration of BMSCs in the damaged brain area, increased the number of tyrosine hydroxylase positive neurons in substantia nigra and striatum, and improved symptomatology [90].

In the same year, other authors, by using a rat model of PD, demonstrated that exercise training might prevent and reduce the disease symptoms [91]. These results came from evidence showing that upon exposure to dopamine toxins to induce PD, the levels of PGC-1α, FNDC5, and BDNF were reduced in the striatum and the hippocampus. Interestingly, in these rats, subjected to treadmill running for 16 weeks before disease induction, the impairments in memory deficits and reduction of biochemical factors (PGC-1α, FNDC5, and BDNF) were prevented [91]. Moreover, in the same animals, behavioral alterations, assessed by spatial learning and memory test, were observed. Taken together, these results suggest that the levels of FNDC5/irisin could play a crucial role in PD symptoms. Several lines of evidence show that exercise may reduce the risk of PD and improve both motor and non-motor symptoms in PD [92]. Recently, physical exercise in midlife has been shown to reduce the prevalence of non-motor symptoms in older individuals, generally associated with PD [93].

Considering the effects of exercise on the onset and clinical development of PD and the increase of irisin after physical activity, it could be of clinical relevance to investigate the effect of irisin administration in a further animal model of PD and in humans in the early stages of the disease.

*Irisin and ALS*. ALS is a progressive neurodegenerative disease involving upper and lower motor neurons. The factors involved in the alteration and cell death are still unclear. However, neuroinflammation seems to be a key factor in the progression of ALS. Indeed, the onset and the pathogenesis of ALS are due to the infiltration of activated microglia and astrocytes that produce a neurotoxic action via the release of pro-inflammatory cytokines [42,94]. At present, very few studies describe the correlation between the expression of FNDC5/irisin and ALS. However, indirect evidence came from the study of Bayer et al., demonstrating that in genetically modified SOD1 mice, the only ALS mouse model currently used, there was an increase of the canonical peripherical PGC-1α system and a reduction in CNS of the specific PGC-1α isoforms [95]. These findings indicate that alteration in the PGC-1α system could be involved in the neurodegeneration of ALS.

Importantly PGC-1α is one of the main regulators of FNDC5 expression [1]. Therefore, the relationship between PGC-1α and neurodegeneration in SOD1 could involve the alteration in the expression of FNDC5/irisin in muscle and/or in neurons.

**Figure 2:**
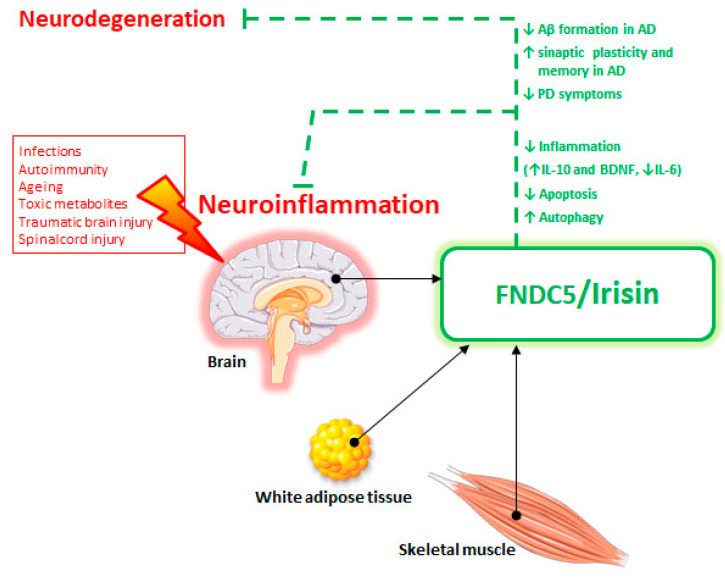
Scheme of fibronectin type III domain-containing protein 5 (FNDC5)/irisin system in the protection against neuroinflammation and neurodegeneration. Black arrows indicate irisin sources. Dotted green lines show the beneficial irisin effects. Green arrows indicate: ↑ increase and ↓ reduction.

In a human study, more recently, it has been shown that in ALS patients, with alteration of the metabolic status, serum irisin levels were upregulated with respect to normo-metabolic ALS patients and control subjects [96]. Studies proving a direct involvement of FNDC5 in ALS are, at present, lacking. However, this is an interesting topic because patients with ALS are characterized by muscle damage, and FNDC5/irisin, known for its anabolic action on skeletal muscle, could represent a biomarker of muscle damage in neurodegenerative diseases (Figure 2).

## 4. Conclusions and Perspective

The claim that physical exercise and well-being state are closely coupled, with the discovery of irisin in 2012, has allowed to actually recognize that irisin can be the molecular mediator of the multiple positive effects on different tissues and organs, including brain health. Starting from the detection of the first effect on adipose tissue, consisting of the induction of browning of white adipocytes, followed by the important anabolic effect on bone and the impact on glucose and lipid metabolism, in vitro and in vivo studies have highlighted the protective effects of irisin on neuroinflammation and neurodegeneration. Recent studies have provided evidence that FNDC5/irisin, through the activation of anti-apoptotic, anti-senescence, and anti-inflammatory pathways, targets different tissues and organs. Interestingly, FNDC5/irisin also sustains neuron survival and function, improving memory and cognition processes and in an animal model of neurodegenerative diseases. The ability of irisin to modulate in the hippocampus the expression of BDNF and microglial activation has provided evidence of irisin involvement in neuroinflammatory responses and neurodegeneration. Ongoing research demonstrating irisin-induced autophagy, as a strategy for protecting cell function, add to the other molecular mechanisms by which irisin can exert its anti-inflammatory effects. Even if in limited number, current studies in animal models of neurodegenerative diseases have shown that irisin administration is effective in preventing or mitigating the development of the disease. Interestingly, some evidence, although still few, support the importance of irisin in human neurodegenerative diseases, suggesting that what has been demonstrated in animal models could be confirmed in humans. To reach this aim, further studies are needed to consider the future therapeutic usage of irisin in neuroinflammation and neurodegenerative diseases.

## 5. Patents

M.G. and S.C. are inventors of the patent titled “Irisin for care and prevention of Osteoporosis”, Italian patent n° 0001429474, granted on 16.08.2017; EU patent n° EP3081228B1, granted on 19.09.2018; USA patent n° US10576127B2, granted on 03.03.2020.

## Data Availability

Not applicable.

## Abbreviations

ND	Neurodegenerative diseases
FNDC5	Fibronectin type III domain-containing protein 5
PeP	Peroxisomal protein
FRCP2	Fibronectin type III repeat-containing protein 2
PGC-1 α	PPARγ co-activator-1α
CSF	Cerebrospinal fluid
UCP1	Uncoupling protein 1
MAPK	Mitogen-activated protein kinases
AMPK	5′ adenosine monophosphate-activated protein kinase
PI3K	Phosphatidylinositol 3-kinase
AKT	Protein kinase B
STAT3	Signal transducer and activator of transcription 3
cAMP	Cyclic adenosine 3′,5′-monophosphate
PKA	Protein kinase A
CREB	cAMP response element-binding protein
Mtss1L	Metastasis-suppressor 1-like
CNS	Central nervous system
PRRS	Pattern-recognition receptors
PAMPs	Pathogen-associated molecular patterns
DAMPs	Damage–associated molecular patterns
IL	Interleukin
TNF	Tumor necrosis factor
ROS	Reactive-oxygen species
NO	Nitric oxide
TLRs	Toll-like receptors
IFN-γ	Interferon-γ
AD	Alzheimer’s disease
PD	Parkinson’s disease
BDNF	Brain-derived neurotrophic factor
TrkB	Tropomyosin receptor kinase B
ERK	Extracellular-signal-regulated kinase
NF-κB	Nuclear factor-kappa light chain enhancer of activated B cells
GSK-3	Glycogen synthase kinase 3
MKP-1	Mitogen-activated protein kinase phosphatase 1
JNK	c-Jun N-terminal kinase
NLRP3	Nod-like receptor pyrin-3
COX-2	Cyclooxygenase-2
Opa1	Optic atrophy 1
ALS	Amyotrophic lateral sclerosis
pTau	Hyperphosphorylated tau
Aβ	Amyloid-beta
APP	Amyloid precursor protein
BACE1	Beta-site amyloid precursor protein cleaving enzyme 1
MPTP	1-methyl-4-phenyl-1,2,3,6-tetrahydropyridine
BMSCs	Bone marrow stromal cells

## References

[1] Boström P., Wu J., Jedrychowski M.P., Korde A., Ye L., Lo J.C., Rasbach K.A., Boström E.A., Choi J.H., Long J.Z. (2012). A PGC1-alpha-dependent myokine that drives brown-fat-like development of white fat and thermogenesis. Nature.

[2] Ferrer-Martínez A., Ruiz-Lozano P., Chien K.R. (2002). Mouse PeP: A novel peroxisomal protein linked to myoblast differentiation and development. Dev. Dyn..

[3] Teufel A., Malik N., Mukhopadhyay M., Westphal H. (2002). Frcp1 and Frcp2, two novel fibronectin type III repeat containing genes. Gene.

[4] Jedrychowski M.P., Wrann C.D., Paulo J.A., Gerber K.K., Szpyt J., Robinson M.M., Sreekumaran Nair K., Gygi S.P., Spiegelman B.M. (2015). Detection and quantitation of circulating human irisin by tandem mass spectrometry. Cell Metab..

[5] Raschke S., Elsen M., Gassenhuber H., Sommerfeld M., Schwahn U., Brockmann B., Jung R., Wisløff U., Tjønna A.E., Raastad T. (2013). Evidence against a beneficial effect of irisin in humans. PLoS ONE.

[6] Albrecht E., Norheim F., Thiede B., Holen T., Ohashi T., Schering L., Lee S., Brenmoehl J., Thomas S., Drevon C.A. (2015). Irisin—A myth rather than an exercise- inducible myokine. Sci. Rep..

[7] Ghahrizjani F.A., Ghaedi K., Salamian A., Tanhaei S., Nejati A.S., Salehi H., Nabiuni M., Baharvand H., Nasr-Esfahani M.H. (2015). Enhanced expression of FNDC5 in human embryonic stem cell-derived neural cells along with relevant embryonic neural tissues. Gene.

[8] Dun S.L., Lyu R.M., Chen Y.H., Chang J.K., Luo J.J., Dun N.J. (2013). Irisin-immunoreactivity in neural and non-neural cells of the rodent. Neuroscience.

[9] Varela-Rodriguez B.M., Pena-Bello L., Juiz-Valina P., Vidal-Bretal B., Cordido F., Sangiao-Alvarellos S. (2016). FNDC5 expression and circulating irisin levels are modified by diet and hormonal conditions in hypothalamus, adipose tissue and muscle. Sci. Rep..

[10] Wrann C.D., White J.P., Salogiannnis J., Laznik-Bogoslavski D., Wu J., Ma D., Lin J.D., Greenberg M.E., Spiegelman B.M. (2013). Exercise induces hippocampal BDNF through a PGC-1alpha/FNDC5 pathway. Cell Metab..

[11] Mahgoub M.O., D’Souza C., Al Darmaki R.S.M.H., Baniyas M.M.Y.H., Adeghate E. (2018). An update on the role of irisin in the regulation of endocrine and metabolic functions. Peptides.

[12] Korta P., Pocheć E., Mazur-Biały A. (2019). Irisin as a multifunctional protein: Implications for health and certain diseases. Medicina.

[13] Colaianni G., Storlino G., Sanesi L., Colucci S., Grano M. (2020). Myokines and osteokines in the pathogenesis of muscle and bone diseases. Curr. Osteoporos. Rep..

[14] Young M.F., Valaris S., Wrann C.D. (2019). A role for FNDC5/Irisin in the beneficial effects of exercise on the brain and in neurodegenerative diseases. Prog. Cardiovasc. Dis..

[15] Colaianni G., Errede M., Sanesi L., Notarnicola A., Celi M., Zerlotin R., Storlino G., Pignataro P., Oranger A., Pesce V. (2020). Irisin correlates positively with BMD in a cohort of older adult patients and downregulates the senescent marker p21 in osteoblasts. J. Bone Min. Res.

[16] Piya M.K., Harte A.L., Sivakumar K., Tripathi G., Voyias P.D., James S., Sabico S., Al-Daghri N.M., Saravanan P., Barber T.M. (2014). The identification of irisin in human cerebrospinal fluid: Influence of adiposity, metabolic markers, and gestational diabetes. Am. J. Physiol. Endocrinol. Metab..

[17] Ruan Q., Zhang L., Ruan J., Zhang X., Chen J., Ma C., Yu Z. (2018). Detection and quantitation of irisin in human cerebrospinal fluid by tandem mass spectrometry. Peptides.

[18] Ruan Q., Huang Y., Yang L., Ruan J., Gu W., Zhang X., Zhang Y., Zhang W., Yu Z. (2019). The effects of both age and sex on irisin levels in paired plasma and cerebrospinal fluid in healthy humans. Peptides.

[19] Lee H.J., Lee J.O., Kim N., Kim J.K., Kim H.I., Lee Y.W., Kim S.J., Choi J.-I., Oh Y., Kim J.H. (2015). Irisin, a novel myokine, regulates glucose uptake in skeletal muscle cells via AMPK. Mol. Endocrinol..

[20] So W.Y., Leung P.S. (2016). Irisin ameliorates hepatic glucose/lipid metabolism and enhances cell survival in insulin-resistant human HepG2 cells through adenosine monophosphate-activated protein kinase signaling. Int. J. Biochem. Cell. Biol..

[21] Xin C., Liu J., Zhang J.D., Zhu D., Wang H., Xiong L., Lee Y., Ye J., Lian K., Xu C. (2016). Irisin improves fatty acid oxidation and glucose utilization in type 2 diabetes by regulating the AMPK signaling pathway. Int. J. Obes. (Lond.).

[22] Arhire L.I., Mihalache L., Covasa M. (2019). Irisin: A hope in understanding and managing obesity and metabolic syndrome. Front. Endocrinol. (Lausanne).

[23] Colaianni G., Cuscito C., Mongelli T., Pignataro P., Buccoliero C., Liu P., Lu P., Sartini L., Di Comite M., Mori G. (2015). The myokine irisin increases cortical bone mass. Proc. Natl. Acad. Sci. USA.

[24] Colaianni G., Mongelli T., Cuscito C., Pignataro P., Lippo L., Spiro G., Notarnicola A., Severi I., Passeri G., Mori G. (2017). Irisin prevents and restores bone loss and muscle atrophy in hind-limb suspended mice. Sci. Rep..

[25] Colucci S., Colaianni G., Brunetti G., Ferranti F., Mascetti G., Mori G., Grano M. (2020). Irisin prevents microgravity-induced impairment of osteoblast differentiation in vitro during the space flight CRS-14 mission. Faseb J..

[26] Storlino G., Colaianni G., Sanesi L., Lippo L., Brunetti G., Errede M., Colucci S., Passeri G., Grano M. (2020). Irisin prevents disuse-induced osteocyte apoptosis. J. Bone Min. Res..

[27] Kim H., Wrann C.D., Jedrychowski M., Vidoni S., Kitase Y., Nagano K., Zhou C., Chou J., Parkman V.A., Novick S.J. (2018). Irisin mediates effects on bone and fat via alphaV integrin receptors. Cell.

[28] Rabiee F., Lachinani L., Ghaedi S., Nasr-Esfahani M.H., Megraw T.L., Ghaedi K. (2020). New insights into the cellular activities of Fndc5/Irisin and its signaling pathways. Cell Biosci..

[29] Moon H.-S., Dincer F., Mantzoros C.S. (2013). Pharmacological concentrations of irisin increase cell proliferation without influencing markers of neurite outgrowth and synaptogenesis in mouse H19-7 hippocampal cell lines. Metabolism.

[30] Lourenco M.V., Frozza R.L., de Freitas G.B., Zhang H., Kincheski G.C., Ribeiro F.C., Gonçalves R.A., Clarke J.R., Beckman D., Staniszewski A. (2019). Exercise-linked FNDC5/irisin rescues synaptic plasticity and memory defects in Alzheimer’s models. Nat. Med..

[31] Kim S., Choi J.-Y., Moon S., Park D.-H., Kwak H.-B., Kang J.-H. (2019). Roles of myokines in exercise-induced improvement of neuropsychiatric function. Pflug. Arch.

[32] Cotman C.W., Berchtold N.C., Christie L.A. (2007). Exercise builds brain health: Key roles of growth factor cascades and inflammation. Trends Neuro Sci..

[33] Mattson M.P. (2012). Energy intake and exercise as determinants of brain health and vulnerability to injury and disease. Cell. Metab..

[34] Voss M.W., Soto C., Yoo S., Sodoma M., Vivar C., van Praag H. (2019). Exercise and Hippocampal Memory Systems. Trends Cogn. Sci..

[35] Chatzi C., Zhang Y., Hendricks W.D., Chen Y., Schnell E., Goodman R.H., Westbrook G.L. (2019). Exercise-induced enhancement of synaptic function triggered by the inverse BAR protein, Mtss1L. Elife.

[36] Warburton D.E.R., Nicol C.W., Bredin S.S.D. (2006). Health benefits of physical activity: The evidence. CMAJ.

[37] Erickson K.I. (2013). Physical activity and brain plasticity in late adulthood. Dialogues Clin. Neurosci..

[38] DiSabato D.J., Quan N., Godbout J.P. (2016). Neuroinflammation: The devil is in the details. J. Neurochem..

[39] Colonna M., Butovsky O. (2017). Microglia function in the central nervous system during health and neurodegeneration. Annu. Rev. Immunol..

[40] Bilbo S., Stevens B. (2017). Microglia: The brain’s first responders. Cerebrum.

[41] Salter M.W., Stevens B. (2017). Microglia emerge as central players in brain disease. Nat. Med..

[42] Kwon H.S., Koh S.H. (2020). Neuroinflammation in neurodegenerative disorders: The roles of microglia and astrocytes. Transl. Neurodegener..

[43] Colombo E., Farina C. (2016). Astrocytes: Key regulators of neuroinflammation. Trends Immunol..

[44] Farina C., Aloisi F., Meinl E. (2007). Astrocytes are active players in cerebral innate immunity. Trends Immunol..

[45] Liddelow S.A., Guttenplan K.A., Clarke L.E., Bennett F.C., Bohlen C.J., Schirmer L., Bennett M.L., Münch A.E., Chung W.S., Peterson T.C. (2017). Neurotoxic reactive astrocytes are induced by activated microglia. Nature.

[46] Liddelow S.A., Marsh S.E., Stevens B. (2020). Microglia and astrocytes in disease: Dynamic duo or partners in crime?. Trends Immunol..

[47] Mee-inta O., Zhao Z.-W., Kuo Y.-M. (2019). Physical Exercise Inhibits Inflammation and Microglial Activation. Cells.

[48] Fischer C.P. (2006). Interleukin-6 in acute exercise and training: What is the biological relevance?. Exerc. Immunol. Rev..

[49] Steensberg A., Fischer C.P., Keller C., Møller K., Pedersen B.K. (2003). IL-6 enhances plasma IL-1ra, IL-10, and cortisol in humans. Am. J. Physiol. Endocrinol. Metab..

[50] Hirsch E.C., Hunot S. (2009). Neuroinflammation in Parkinson’s disease: A target for neuroprotection?. Lancet Neurol..

[51] Scheller J., Chalaris A., Schmidt-Arras D., Rose-John S. (2011). The pro- and anti-inflammatory properties of the cytokine interleukin-6. Biochim. Biophys. Acta.

[52] Gabay C. (2006). Interleukin-6 and chronic inflammation. Arthritis Res..

[53] Singh-Manoux A., Dugravot A., Brunner E., Kumari M., Shipley M., Elbaz A., Kivimaki M. (2014). Interleukin-6 and C-reactive protein as predictors of cognitive decline in late midlife. Neurology.

[54] Pedersen B.K. (2012). Muscular interleukin-6 and its role as an energy sensor. Med. Sci. Sports Exerc..

[55] Dong Y., Pu K., Duan W., Chen H., Chen L., Wang Y. (2018). Involvement of Akt/CREB signaling pathways in the protective effect of EPA against interleukin-1beta-induced cytotoxicity and BDNF down-regulation in cultured rat hippocampal neurons. BMC Neurosci..

[56] Qin L., Bouchard R., Pugazhenthi S. (2016). Regulation of cyclic AMP response element-binding protein during neuroglial interactions. J. Neurochem..

[57] Li X., Jope R.S. (2010). Is glycogen synthase kinase-3 a central modulator in mood regulation?. Neuropsychopharmacology.

[58] Suri D., Vaidya V.A. (2013). Glucocorticoid regulation of brain-derived neurotrophic factor: Relevance to hippocampal structural and functional plasticity. Neuroscience.

[59] Jeanneteau F., Deinhardt K., Miyoshi G., Bennett A.M., Chao M.V. (2010). The MAP kinase phosphatase MKP-1 regulates BDNF-induced axon branching. Nat. Neurosci..

[60] Littlefield A.M., Setti S.E., Priester C., Kohman R.A. (2015). Voluntary exercise attenuates LPS-induced reductions in neurogenesis and increases microglia expression of a proneurogenic phenotype in aged mice. J. Neuroinflamm..

[61] Li D.J., Li Y.H., Yuan H.B., Qu L.F., Wang P. (2017). The novel exercise-induced hormone irisin protects against neuronal injury via activation of the Akt and ERK1/2 signaling pathways and contributes to the neuroprotection of physical exercise in cerebral ischemia. Metabolism.

[62] Peng J., Deng X., Huang W., Yu J.H., Wang J.X., Wang J.P., Yang S.B., Liu X., Wang L., Zhang Y. (2017). *Irisin* protects against neuronal injury induced by oxygen-glucose deprivation in part depends on the inhibition of ROS-NLRP3 inflammatory signaling pathway. Mol. Immunol..

[63] Dameni S., Janzadeh A., Yousefifard M., Nasirinezhad F. (2018). The effect of intrathecal injection of irisin on pain threshold and expression rate of GABAB receptors in peripheral neuropathic pain model. J. Chem. Neuroanat..

[64] Wang K., Li H., Wang H., Wang J.-H., Song F., Sun Y. (2018). Irisin Exerts Neuroprotective Effects on Cultured Neurons by Regulating Astrocytes. Mediat. Inflamm.

[65] Pesce M., Ballerini P., Paolucci T., Puca I., Farzaei M.H., Patruno A. (2020). Irisin and Autophagy: First Update. Int. J. Mol. Sci..

[66] Rocchi A., Yamamoto S., Ting T., Fan Y., Sadleir K., Wang Y., Zhang W., Huang S., Levine B., Vassar R. (2017). A Becn1 mutation mediates hyperactive autophagic sequestration of amyloid oligomers and improved cognition in Alzheimer’s disease. PLoS Genet..

[67] Xin T., Lu C. (2020). Irisin activates Opa1-induced mitophagy to protect cardiomyocytes against apoptosis following myocardial infarction. Aging (Albany Ny).

[68] Kempuraj D., Thangavel R., Natteru P.A., Selvakumar G.P., Saeed D., Zahoor H., Zaheer S., Iyer S.S., Zaheer A. (2016). Neuroinflammation induces neurodegeneration. J. Neurol. Neurosurg. Spine.

[69] Li Z., Zheng Z., Ruan J., Li Z., Tzeng C.M. (2016). Chronic inflammation links cancer and Parkinson’s disease. Front. Aging Neurosci..

[70] Wyss-Coray T., Mucke L. (2002). Inflammation in neurodegenerative disease—A double-edged sword. Neuron.

[71] Askari H., Rajani S.F., Poorebrahim M., Haghi-Aminjan H., Raeis-Abdollahi E., Abdollahi M. (2018). A glance at the therapeutic potential of irisin against diseases involving inflammation, oxidative stress, and apoptosis: An introductory review. Pharm. Res..

[72] Jiang X., Shen Z., Chen J., Wang C., Gao Z., Yu S., Yu X., Chen L., Xu L., Chen Z. (2020). Irisin protects against motor dysfunction of rats with spinal cord injury via Adenosine 5′-Monophosphate (AMP)-activated protein kinase-nuclear factor kappa-B pathway. Front. Pharmacol..

[73] Ferreira S.T., Lourenco M.V., Oliveira M.M., De Felice F.G. (2015). Soluble amyloid-β oligomers as synaptotoxins leading to cognitive impairment in Alzheimer’s disease. Frontiers in Cellular Neuroscience.

[74] Selkoe D.J. (2002). Alzheimer’s disease is a synaptic failure. Science.

[75] Lepeta K., Lourenco M.V., Schweitzer B.C., Martino Adami P.V., Banerjee P., Catuara-Solarz S. (2016). Synaptopathies: Synaptic dysfunction in neurological disorders. J. Neurochem..

[76] Kang J., Lemaire H.G., Unterbeck A., Salbaum J.M., Masters C.L., Grzeschik K.H., Multhaup G., Beyreuther K., Müller-Hill B. (1987). The precursor of Alzheimer’s disease amyloid A4 protein resembles a cell-surface receptor. Nature.

[77] De Felice F.G., Vieira M.N., Saraiva L.M., Figueroa-Villar J.D., Garcia-Abreu J., Liu R., Chang L., Klein W.L., Ferreira S.T. (2004). Targeting the neurotoxic species in Alzheimer’s disease: Inhibitors of Abeta oligomerization. STFASEB J..

[78] Gralle M., Ferreira S.T. (2007). Structure and functions of the human amyloid precursor protein: The whole is more than the sum of its parts. Prog. Neurobiol..

[79] Vassar R. (2004). BACE1: The beta-secretase enzyme in Alzheimer’s disease. J. Mol. Neurosci..

[80] Holsinger R.M., McLean C.A., Beyreuther K., Masters C.L., Evin G. (2002). Increased expression of the amyloid precursor beta-secretase in Alzheimer’s disease. Ann. Neurol.

[81] Cole S.L., Vassar R. (2008). The role of amyloid precursor protein processing by BACE1, the beta-secretase, in Alzheimer disease pathophysiology. J. Biol. Chem..

[82] Noda Y., Kuzuya A., Tanigawa K., Araki M., Kawai R., Ma B., Sasakura Y., Maesako M., Tashiro Y., Miyamoto M. (2018). Fibronectin type III domain-containing protein 5 interacts with APP and decreases amyloid β production in Alzheimer’s disease. Mol. Brain.

[83] Lourenco M.V., Ribeiro F.C., Sudo F.K., Drummond C., Assunção N., Vanderborght B., Tovar-Moll F., Mattos P., De Felice F.G., Ferreira S.T. (2020). Cerebrospinal fluid irisin correlates with amyloid-β, BDNF, and cognition in Alzheimer’s disease. Alzheimers Dement. (Amst)..

[84] Küster O.C., Laptinskaya D., Fissler P., Schnack C., Zügel M., Nold V., Thurm F., Pleiner S., Karabatsiakis A., von Einem B. (2017). Novel Blood-Based Biomarkers of Cognition, Stress, and Physical or Cognitive Training in Older Adults at Risk of Dementia: Preliminary Evidence for a Role of BDNF, Irisin, and the Kynurenine Pathway. J. Alzheimers Dis..

[85] Tsai C.L., Pai M.C. (2020). Circulating levels of Irisin in obese individuals at genetic risk for Alzheimer’s disease: Correlations with amyloid-β, metabolic, and neurocognitive indices. Behav. Brain Res..

[86] Petersen R.C., Caracciolo B., Brayne C., Gauthier S., Jelic V., Fratiglioni L. (2014). Mild cognitive impairment: A concept in evolution. J. Intern. Med..

[87] Jessen F., Amariglio R.E., van Boxtel M., Breteler M., Ceccaldi M., Chételat G., Dubois B., Dufouil C., Ellis K.A., van der Flier W.M. (2014). Subjective Cognitive Decline Initiative (SCD-I) Working Group. A conceptual framework for research on subjective cognitive decline in preclinical Alzheimer’s disease. Alzheimers Dement..

[88] Jack C.R., Bennett D.A., Blennow K., Carrillo M.C., Dunn B., Haeberlein S.B., Holtzman D.M., Jagust W., Jessen F., Karlawish J. (2018). Contributors. NIA-AA Research Framework: Toward a biological definition of Alzheimer’s disease. Alzheimer Dement..

[89] Auluck P.K., Chan H.Y., Trojanowski J.Q., Lee V.M., Bonini N.M. (2002). Chaperone suppression of alpha-synuclein toxicity in a Drosophila model for Parkinson’s disease. Science.

[90] Zarbakhsh S., Safari M., Aldaghi M.R., Sameni H.R., Ghahari L., Khaleghi Lagmouj Y., Rahimi Jaberi K., Parsaie H. (2019). Irisin protects the substantia nigra dopaminergic neurons in the rat model of Parkinson’s disease. Iran. J. Basic Med. Sci..

[91] Rezaee Z., Marandi S.M., Alaei H., Esfarjani F. (2019). The effect of preventive exercise on the neuroprotection in 6-hydroxydopamine-lesioned rat brain. Appl. Physiol. Nutr. Metab..

[92] Xu X., Fu Z., Le W. (2019). Exercise and Parkinson’s disease. Int. Rev. Neurobiol..

[93] Hughes K.C., Gao X., Molsberry S., Valeri L., Schwarzschild M.A., Ascherio A. (2019). Physical activity and prodromal features of Parkinson disease. Neurology.

[94] Liu J., Wang F. (2017). Role of Neuroinflammation in Amyotrophic Lateral Sclerosis: Cellular Mechanisms and Therapeutic Implications. Front. Immunol..

[95] Bayer H., Lang K., Buck E., Higelin J., Barteczko L., Pasquarelli N., Sprissler J., Lucas T., Holzmann K., Demestre M. (2017). ALS-causing mutations differentially affect PGC-1α expression and function in the brain vs. peripheral tissues. Neurobiol. Dis..

[96] Lunetta C., Lizio A., Tremolizzo L., Ruscica M., Macchi C., Riva N., Sansone V. (2018). Serum irisin is upregulated in patients affected by amyotrophic lateral sclerosis and correlates with functional and metabolic status. J. Neurol..

